# The Effect of Melatonin Supplementation on Cancer-Related Fatigue during Chemotherapy Treatment of Breast Cancer Patients: A Double-Blind, Randomized Controlled Study

**DOI:** 10.3390/cancers16040802

**Published:** 2024-02-16

**Authors:** Frantzeska Nimee, Aristea Gioxari, Panos Papandreou, Charalampia Amerikanou, Sofia Karageorgopoulou, Andriana C. Kaliora, Maria Skouroliakou

**Affiliations:** 1 Department of Dietetics and Nutritional Science, School of Health Science and Education, Harokopio University, 70 El. Venizelou Ave., 17676 Athens, Greece; fnimee@hua.gr (F.N.); amerikanou@windowslive.com (C.A.); mskour@hua.gr (M.S.); 2 Department of Nutritional Science and Dietetics, School of Health Sciences, 24100 Kalamata, Greece; a.gioxari@uop.gr; 3 Department of Nutrition, IASO Hospital, 37 Chomatianou Str., Marousi, 15123 Athens, Greece; ppapandreou@cibusmed.com; 4 Third Department of Medical Oncology, IASO Hospital, 37 Chomatianou Str., Marousi, 15123 Athens, Greece; skarageorg@hotmail.com

**Keywords:** melatonin, supplement, cancer-related fatigue, breast cancer, chemotherapy

## Abstract

**Simple Summary:**

Cancer-related fatigue is a common distressing complaint of breast cancer patients treated with chemotherapy. Nutritional quality plays a pivotal role in cancer-related fatigue, while increased interest towards new pharmacological agents has been observed. Melatonin, an endogenous hormone that regulates the human sleep–wake cycle, could alleviate cancer-related fatigue. In the present human trial, we investigated the effects of melatonin intake (i.e., 1 mg/day) vs. placebo on fatigue in women with active breast cancer over a period of 3 months. In both groups, nutritional advice regarding the Mediterranean diet was implemented. At the trial endpoint, the results showed that only patients receiving melatonin improved cancer-related fatigue compared to baseline. This implies that the oral supplementation of melatonin could ameliorate fatigue in breast cancer patients who undergo chemotherapy treatment.

**Abstract:**

Cancer-related fatigue (CRF) is a common distressing complaint of breast cancer (BC) patients treated with chemotherapy. Nutritional quality plays a pivotal role in CRF, while increased interest towards new pharmacological agents has been observed. Melatonin, an endogenous hormone that regulates the human sleep–wake cycle, could alleviate CRF. In the present randomized, placebo-controlled 3-month trial, we investigated the effects of melatonin intake (i.e., 1 mg/day) vs. placebo in BC patients on CRF. In both arms, the Mediterranean diet (MD) was implemented. Medical history, anthropometry and blood withdrawal were performed. CRF was evaluated by the Functional Assessment of Chronic Illness Therapy—Fatigue questionnaire and MD adherence by the MedDietScore. In total, 49 BC women (median age 52 years) were recruited, namely N = 23 in the intervention arm and N = 26 in the placebo arm. At baseline, CRF was positively associated with body mass index (BMI), even when adjusted for age, waist circumference and blood indices related to disease prognosis (beta = −0.882, *p* = 0.003). At 3 months, both groups showed a BMI decrease (*p* < 0.05), but only the intervention group improved CRF compared to baseline (*p* = 0.003). No differences in CRF were observed between the groups. In conclusion, melatonin oral supplementation could ameliorate CRF in BC patients.

## 1. Introduction

Breast cancer (BC) is the most commonly diagnosed cancer among women [1]. In 2020, over 2 million new cases of BC were reported, surpassing those of lung cancer [1]. Chemotherapy is a usual treatment option, but health-related adverse effects have been commonly reported, including cancer-related fatigue (CRF) [2]. The National Comprehensive Cancer Network (NCCN) defined CRF as a “distressing, persistent, subjective sense of physical, emotional, and/or cognitive tiredness or exhaustion related to cancer and/or cancer treatment that is not proportional to recent activity, and interferes with usual functioning” [2]. CRF has been characterized as one of the most distressing complaints, while up to 80% of patients treated with chemotherapy experience fatigue [3]. With regard to BC, the incidence of CRF during treatment varies from 28% to 91% [4]. In addition, there is evidence that female cancer patients experience more side effects due to cancer treatment than their male counterparts, demonstrating the need to design more effective methods to combat chemotherapy side effects, especially in women [5].

The symptomatology of CRF includes physical fatigue, stress, attention deficit, mental fatigue, nausea, short-term memory loss, physical pain, and lack of concentration [6]. There is a substantially negative impact on work, social relationships, mood, and daily activities, resulting in a significant deterioration of health-related quality of life pre- and post-treatment [7]. During the last few decades, a plethora of pharmacological and non-pharmacological interventions have led to controversial results regarding fatigue management [8]. Consequently, there is no gold-standard method for treating CRF.

The pathophysiology of CRF is still unknown. Several physiological factors have been investigated for fatigue development in cancer, i.e., neuropsychological impairment, muscle metabolism dysregulation, disruption of circadian rhythms, immune activation, oxidative stress, hormonal changes affecting the hypothalamic–pituitary axis, and premature menopause [6,7]. The NCCN panel has also identified several environmental factors that may contribute to CRF, such as nutritional quality, alcohol/substance abuse, as well as individual factors including emotional distress, poor sleep hygiene and sleep disturbance [2]. To this point, sleeping agents could serve as a potential therapy for CRF [9]. Pharmacological agents include benzodiazepine receptor agonists, benzodiazepines, antihistamines, modafinil, anxiolytics and antidepressants [10,11,12], but these have been documented to exert several side effects, such as hypersomnia, cognitive disabilities, drug dependence, tolerance, and accidents/falls [12].

To overcome this, the medical community has started intensive research towards effective treatments with fewer health complications. For instance, the hormone melatonin, which is naturally secreted by the pineal gland, has been extensively studied for its effects on sleep quality upon exogenous administration [13]. Endogenous melatonin is secreted into the blood circulation at night following the circadian rhythm. Melatonin regulates the human sleep–wake cycle by acting on MT1 and MT2 receptors in the suprachiasmatic nucleus of the hypothalamus [13,14]. As a result, the augmentation of blood melatonin begins at about two hours before sleep and the highest concentrations are observed five hours later [15]. The exogenous administration of melatonin in animal models seems to exert favorable effects on sleep onset time and sleep duration [14,16]. In humans, oral melatonin treatment has been reported to be safe and well tolerated, showing low dependence and a limited drug interaction profile [17]. According to recent meta-analyses of randomized controlled trials (RCTs), melatonin oral supplementation was associated with improved sleep quality and sleep onset latency [18,19]. The role of melatonin in cancer prognosis has been extensively studied. The outcomes of a recent meta-analysis of eight RCTs showed that melatonin intake led to improvements in tumor remission, 1-year survival, and alleviation of radiochemotherapy-related side effects [20]. Nevertheless, the potential effect of melatonin on breast CRF has not been fully explored.

As regards nutritional quality, it is now decisively confirmed that adherence to the Mediterranean diet (MD) is associated with a lower risk of BC development [21,22] and a greater health-related quality of life among BC patients [23,24]. One possible underlying mechanism is the high antioxidant potential of the plant-based Mediterranean dietary pattern, which is rich antioxidants and anti-inflammatory nutrients including mono-unsaturated fatty acids, phenolic compounds, and antioxidant vitamins [22,25].

Therefore, the aim of the present 3-month randomized, placebo-controlled trial was to evaluate whether adherence to the MD along with melatonin supplementation or adherence to the MD with placebo would ameliorate CRF in BC patients receiving chemotherapy treatment.

## 2. Materials and Methods

### 2.1. Ethics and Participants

The Ethics Committee of “IASO Hospital” (Athens, Greece) approved the trial protocol as indicated by the Approval Code #D231052019. The study was performed in accordance with the principles of the Helsinki Declaration (1964) and the terms of Good Clinical Practice. Throughout the trial, the General Data Protection Regulation (EU) 2016/679 was adhered to. Registration with ClinicalTrials.gov (assessed on 1 January 2022) was also acquired (#NCT 01052022).

Adult BC patients of the Oncology Center at IASO Hospital (Athens, Greece) were invited to take part in the study through posters, website and social media announcements. Patients who responded to invitations received a detailed information document describing the aim, methodology, benefits and potential hazards of the study. A written informed consent form was handed to all participants, and each recruited patient kept a copy of the signed consent form. All eligible participants were recruited in January 2022, while the intervention period lasted 3 months, up to May 2022.

The inclusion criteria were set as follows:

(a) Adult women (≥18 years of age) with a BC diagnosis who underwent chemotherapy at the time of invitation;
(b) Women receiving pharmacological treatment not interacting with melatonin;
(c) Women receiving standard pharmacological treatment for at least last two months prior to the study initiation;
(d) Patients with good performance status, as indicated by scoring “0 or 1” on the Eastern Cooperative Oncology Performance Status (ECOG PS) questionnaire [26];
(e) Patients not needing a transfusion, as indicated by hemoglobin ≥9 g/dL;
(f) Patients having the ability to understand and give a written statement of consent.

Patients were excluded from the study in the following scenarios:

(g) Fatigue was attributed to conditions other than cancer, e.g., uncontrolled hypothyroidism, hypercalcemia, congestive heart failure, chronic obstructive pulmonary disease;
(h) Using pharmacological agents for CRF or sleeping disorders prior to the study;
(i) Pharmacological treatment that was modified during the study or that could interact with melatonin;
(j) Diagnosed with gastrointestinal diseases that could affect the absorption of nutrients such as inflammatory bowel disease infections;
(k) Diagnosed with psychiatric disorders such as depression, psychosis, and bipolar disorder and receiving equivalent medication;
(l) Excessive alcohol consumption;
(m) A lifestyle that can affect sleep patterns (e.g., night shifts);
(n) Patients with a poor clinical state as indicated by laboratory markers: creatinine clearance <30 mL/min; aspartate aminotransferase (AST) > 3 × upper limit of normal (ULN); alanine aminotransferase (ALT) > 3 × ULN; bilirubin > 1 × ULN.

### 2.2. Study Design and Methods

Study design: In this two-armed, double-blinded, single-center, randomized, placebo-controlled, 3-month trial, eligible BC patients were randomly assigned to either the placebo or the intervention arm. An independent statistician performed a simple randomization sequence using computer software, and blinding of the allocated treatment was maintained throughout the intervention to all researchers and the data analyst. The duration of the intervention for both arms was 3 months. Patients of the intervention group (melatonin group) received a personalized dietary plan based on the Mediterranean diet, together with a booklet of lifestyle guidelines (nutritional and physical activity), all generated by a Clinical Decision Support System (CDSS). A detailed description of the particular CDSS has already been given in our research team’s previous work [23]. Moreover, participants in the intervention group consumed one melatonin tablet per day together with water for a total of three months.

In the placebo group, patients also received a CDSS-derived dietary plan based on the Mediterranean diet together with written lifestyle recommendations. Participants in this group were instructed to consume one placebo tablet (with water) every day for a total of three months.

Melatonin and placebo tablets: The European Commission granted marketing authorization valid throughout the European Union for oral prolonged-release tablets that contain 2 mg of melatonin on 29 June 2007. According to the European Public Assessment Report (updated in May 2010), a daily intake of 2 mg of melatonin is safe for human use for a period of 3 weeks up to 6 months. Side effects are rare, and 1 to 10 patients in 1000 experience marginal symptoms, e.g., irritability, nervousness, restlessness, insomnia, abnormal dreams, anxiety, migraine, lethargy, psychomotor hyperactivity, dizziness, somnolence, and hypertension. The report clarifies that alcohol consumption should be avoided before, during, and after taking melatonin. A melatonin dietary supplement has also gained a health claim by the European Food Safety Authority (EFSA) for the reduction in sleep onset latency (ID 1698, 1780, 4080). According to the EFSA’s scientific opinion, the Panel on Dietetic Products, Nutrition and Allergies concluded that 1 mg of melatonin should be consumed close to bedtime in order to obtain the claimed effect.

In the present study, BC patients of the intervention group received one tablet of dietary supplement with melatonin that was commonly available in local pharmacy stores, daily for 3 months. Each tablet contained 1 mg of melatonin, and patients were instructed to take the tablet one hour before bedtime and two hours after the last meal in order to avoid potential drug–food interactions [27]. Participants of the placebo group consumed a placebo tablet daily following the same instructions. All patients were advised to avoid alcohol consumption during the trial. The melatonin and placebo tablets were of similar physical and sensory properties. For blinding purposes, 30 tablets (verum or placebo) were contained in dark glass bottles and were all of the same brand and batch origin. Uniformity, chemical stability and absence of pathogens per item were assured. The chemical composition of the melatonin and placebo tablets are presented in Table 1.

Medical record: A detailed medical history was obtained for all eligible patients, i.e., type and stage of breast cancer, performed surgery, medication treatment and symptomatology, adherence issues, drug interactions, drug–food interactions, and side effects. Smoking habits, allergies, gastrointestinal problems, or other health issues were recorded for each patient. Additionally, pharmaceutical consultation, including the proper use of medications, the managing of side effects, and guidance on the proper use of over-the-counter (OTC) medications, was offered by licensed pharmacologists. A CDSS that consisted of integrated and evidence-based data on the Greek market medications supported the pharmacists throughout the pharmaceutical care process [28].

Anthropometry assessment: Body weight (BW) and body composition were assessed at the beginning and at the end of the study in both groups. More specifically, BW was measured on a flat scale to the nearest 0.1 kg. Body fat mass (FM) was evaluated with the method of Air Displacement Plethysmography (Bodpod^®^ Body Composition Tracking Systems, Life Measurement, Inc., Rome, Italy). In brief, the examinees performed the test in the morning after overnight fasting and abstaining from rigorous exercise during the previous day.

Participants’ height (Ht) was also measured at the beginning of the study with a calibrated stadiometer to the nearest millimeter (Seca Mode 220, Hamburg, Germany). Body mass index (BMI), defined as the ratio of BW (kg) to the square of Ht (m^2^), was calculated for every volunteer at the start and the end of the study. In addition, waist circumference (WC) was measured with a stretch-resistant measuring tape to the nearest 1 mm at the midpoint between the lower margin of the last palpable rib and the top of the iliac. In total, x3 WC measurements were performed, and the average value was calculated. 

CRF and dietary assessment: CRF was assessed at the beginning and at the end of the study with the Functional Assessment of Chronic Illness Therapy—Fatigue (FACIT-F) questionnaire, which is the most commonly used scale for measuring fatigue of the past seven days [29]. The FACIT-F is a 40-item scale that assesses self-reported fatigue in terms of daily activities and function. It has five subscale domains: physical well-being (PBW), social/family well-being (SFWB), emotional well-being (EWB), functional well-being (FWB), and fatigue. For each question, there are five answers (not at all, a little bit, somewhat, quite a bit, very much). The higher the score, the better the participant’s well-being [30].

Adherence to the MD was assessed by the MedDietScore at the beginning and at the end of the study in both groups. The MedDietScore relies on the amount and frequency of consumed foods that belong to the traditional MD, namely non-refined cereals, potatoes, vegetables, fruits, legumes, fish, and olive oil [31]. Values closer to 55 indicate higher adherence to the MD.

Blood withdrawal and analyses: At baseline and follow-up (3 months), 10 mL of whole blood was withdrawn from each participant after overnight fasting. For serum collection, whole-blood samples were allowed to clot at room temperature for 20 min. The samples were then centrifuged at 3000 rpm for 10 min (4 °C). To isolate plasma from whole blood, tubes containing ethylenediaminetetraacetic acid (EDTA) were used prior to centrifugation. For analyses, freshly drawn samples were used.

Hematology analysis was performed using an automatic analyzer (DxH 800 analyzer, Beckman Coulter Inc., Nyon, Switzerland). Serum glucose (GLU) and lactate dehydrogenase (LDH) were quantified with an automatic biochemical analyzer using manufacturer’s reagents (Cobas 8000 modular analyzer, Roche Diagnostics GmbH, Mannheim, Germany).

### 2.3. Statistical Analysis

The primary outcome of the present study was a significant improvement in FACIT-F total or subscale scores in the intervention vs. placebo groups. To assess variable distribution, the Shapiro–Wilk test was carried out. Categorical variables are expressed as counts (n) and percentages (%), while continuous variables are expressed as median plus interquartile range (IQR). Differences between the intervention and placebo groups at the trial endpoint were assessed by the Mann–Whitney U test. Differences inside a group before and after the intervention were assessed by the Wilcoxon signed-rank test. Linear regression analysis was performed to investigate the possible associations of FACIT-F total score with BMI and MedDietScore after the log transformation of non-normal values. Unadjusted and adjusted models were used, i.e., adjusted model 1: age; adjusted model 2: age, WC; adjusted model 3: age, WC, white blood count (WBC), platelet count (PL), LDH, GLU. All analyses were performed with the SPSS statistical software (version 29.0, SPSS, Inc., ΙΒΜ, Chicago, IL, USA). Statistical significance was set at *p*-value < 0.05.

## 3. Results

### 3.1. Participants and Baseline Characteristics

**Figure 1:**
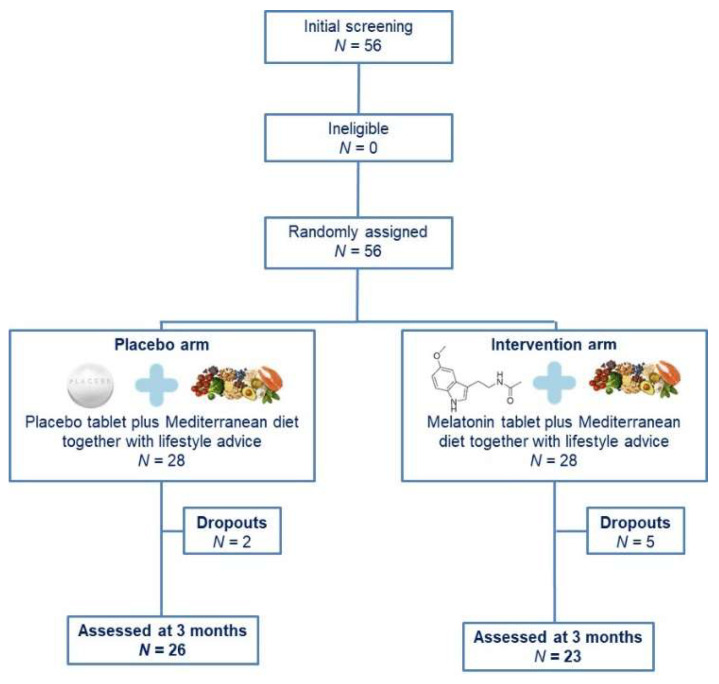
Study flow chart.

Overall, 56 BC patients responded to our invitation and fulfilled the inclusion criteria (Figure 1). Following randomization, 28 women were allocated to each group. Nevertheless, seven patients withdrew from the study: five patients from the intervention group (three for personal reasons and two did not respond to our communication efforts) and two patients from the placebo group (for personal reasons). All other patients completed the study, namely 23 women in the intervention group and 26 in the placebo group. The participants’ nationality was Greek and they were residents of Attica in Greece.

The baseline characteristics per treatment arm are shown in Table 2. The descriptive statistics showed that 36.7% of patients were of normal weight, whereas 59.2% were living with overweight/obesity. In Table 2, the oral drug treatment (other than chemotherapy) is presented according to the Anatomical Therapeutic Chemical (ATC) classification system. No statistically significant differences were found between the placebo and melatonin groups in the mean ranks of anthropometric indices and blood markers, nor in the MedDiet and FACIT-F scores. 

### 3.2. Intra-Group and Inter-Group Comparisons

The differences between the placebo and melatonin groups for all tested characteristics (i.e., anthropometry, blood markers, level of adherence to the Mediterranean diet, and FACIT-F score) are shown in Supplementary Table S1. Compared to baseline, patients in both arms had significantly improved MedDietScores at follow-up [melatonin group: 32.0 (5.0) vs. 34.0 (6.0), *p* = 0.004; placebo group: 34.5 (6.3) vs. 35.0 (5.0), *p* < 0.001], as well as anthropometric markers, i.e., BW [melatonin group: 69.9 kg (22.4) vs. 68.0 kg (19.1), *p* = 0.008; placebo group: 70.7 kg (25.0) vs. 68.5 kg (23.4), *p* < 0.001], BMI [melatonin group: 26.4 kg/m^2^ (8.1) vs. 25.6 kg/m^2^ (6.6), *p* = 0.012; placebo group: 28.3 kg/m^2^ (11.4) vs. 28.0 kg/m^2^ (11.2), *p* < 0.001], FM% [melatonin group: 39.9% (17.7) vs. 39.0% (12.0), *p* = 0.042; placebo group: 44.0% (14.2) vs. 43.0% (13.2), *p* < 0.001], and WC [melatonin group: 96.5 cm (15.0) vs. 94.0 cm (16.5), *p* = 0.005; placebo group: 96 cm (24.3) vs. 95.0 cm (23.3), *p* < 0.001]. However, these variables did not differ between the two groups at 3 months (Supplementary Table). Additionally, blood indices remained unchanged during the trial in both groups (Supplementary Table).

**Figure 2:**
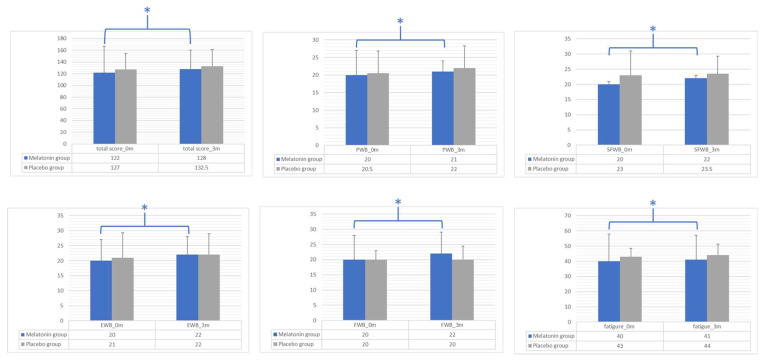
Intra-group and inter-group differences between the melatonin and placebo groups for FACIT-F total score and subscale scores (i.e., PWB, SFWB, EWB, FWB, fatigue). Bar graphs show the median values and interquartile ranges for tested variables at baseline (0 m) and 3 months (3 m) in the melatonin group (blue bars) and the placebo group (gray bars). Differences between the two groups at the trial endpoint were assessed by the Mann–Whitney U test. Differences inside a group before and after the intervention were assessed by the Wilcoxon signed-rank test. * *p*-value: significant differences between baseline and follow-up (3 months) analyzed by the Wilcoxon signed-rank test; statistical significance was set at *p*-value < 0.05. FACIT-F, Functional Assessment of Chronic Illness Therapy—Fatigue; PBW, physical well-being; SFWB, social/family well-being; EWB, emotional well-being; FWB, functional well-being.

A significant increase in FACIT-F and subscale scores (i.e., PWB, SFWB, EWB, FWB, fatigue) was observed in the intervention group at follow-up compared to baseline, but the differences were not significant between the two arms at 3 months (Figure 2).

### 3.3. Linear Regression Analysis

Table 3 presents the outcomes of linear regression models (unadjusted model, adjusted model 1, adjusted model 2, and adjusted model 3) regarding the associations of the FACIT-F total score with BMI and MedDietScore. FACIT-F was inversely and significantly associated with BMI and positively associated with the MedDietScore in adjusted models 1 (age) and 2 (age and WC). When adjustment for blood indices was included (adjusted model 3), the association of FACIT-F with BMI still remained (*p* = 0.003), but this was not the case for MedDietScore (*p* = 0.179).

## 4. Discussion

In the present randomized, placebo-controlled study, we evaluated the effects of melatonin supplementation on CRF in BC patients following chemotherapy. At follow-up (3 months), both groups increased adherence to the Mediterranean dietary pattern compared to baseline, while significant improvements in BW, BMI, body fat mass, and WC were observed. Additionally, patients receiving melatonin supplement (1 mg per day) showed a significant improvement in CRF compared to baseline, as indicated by the FACIT-F scores, although no differences were observed with the placebo group at follow-up.

It is well known that overweight/obesity is a major risk factor for BC development [32]. Overweight/obesity is associated with BC recurrence and poor disease prognosis, contributing to lower overall and breast cancer survival in pre- and postmenopausal women with BC [33,34,35]. Lifestyle interventions including dietary interventions and physical activity regimens have shown to improve body weight status, WC circumference, and quality of life in BC survivors living with overweight/obesity [36]. According to recently published works by our research team, BC patients adhering to the MD for 3 months achieved significant ameliorations in anthropometry markers (i.e., BW, FM%, WC) and blood indices (i.e., fasting GLU and blood lipids) compared to placebo groups. These outcomes were associated with lower oxidative stress, as indicated by significant differences in plasma vitamin C and malondialdehyde levels [23,24]. Additionally, high adherence to the MD is associated with better survival and lower all-cause mortality in BC women [37]. In the present study, both groups, the melatonin and placebo groups, received a personalized dietary regimen based on the MD. At the end of 3 months, both groups had ameliorated BMI, FM%, and WC compared to baseline, while no changes were observed between the two groups. The favorable effects of the MD are probably attributed to its nutrient and non-nutrient content [38]. In the PREDIMED trial, supplementation of the MD with extra virgin olive oil, rich in monounsaturated fatty acids and phytochemicals, was beneficial in the prevention of primary BC in women with an elevated risk of developing cardiovascular diseases [22]. Moreover, elevated consumption of dietary fiber, a major component of the MD, has been strongly associated with a reduction in both all-cause and BC-specific mortality [39].

It is well documented that adherence to the MD improves health-related quality of life in BC women. More specifically, patients following the MD have reported improvements in role functioning and emotional functioning, as well as depression and anxiety [23]. According to the results of a recent randomized trial, cancer patients following the MD for 8 weeks showed a significant amelioration in fatigue, as indicated by FACIT-F scores. This effect was more profound for those patients having lower MedDietScores at baseline [40]. The favorable effects of the MD on CRF have been linked with substantial improvements in mitochondrial function including basal respiration, maximal respiration, and spare capacity [40]. It has been documented that obesity in BC is directly linked with CRF, and this association is probably attributed to the high levels of circulating inflammatory cytokines, i.e., tumor necrosis factor-alpha (TNF-alpha) and interleukin-6 (IL-6), as well as blood fatty acid imbalance [41]. In the present study, we showed that CRF in breast cancer was negatively associated with MedDietScore and positively associated with BMI, even when adjusted for blood marker-related disease prognosis.

In our study, intervention with the MD alone (placebo arm) for 3 months resulted in about 3% weight loss, without any significant effect on CRF. Little is known regarding the optimal weight loss associated with CRF amelioration. According to the LISA trial, a mean 6% weight loss was linked to a more likely preservation of physical condition and improvements in functional and symptom domains [42,43].

According to the results of the present trial, the intake of a melatonin dietary supplement (1 mg/day) for 3 months along with MD adherence led to a significant improvement in CRF. In the study by Innominato and co-authors, 32 patients with metastatic BC took 5 mg of melatonin before bedtime for a total of 2 months. At the end of the intervention, melatonin was associated with a significant improvement in fatigue severity and global quality of life, which were probably attributed to an increase in the expression of the core clock genes, namely genes PER2 and BMAL1 in peripheral blood mononuclear cells [44]. Furthermore, the daily administration of 18 mg of melatonin in BC patients who underwent chemotherapy and radiotherapy significantly improved levels of fatigue compared to the control group [45].

With regard to the effects on mood, melatonin intake for 3 months significantly reduced the risk of post-operative depressive symptoms in BC women recruited for the MELODY randomized, placebo-controlled trial. Moreover, melatonin supplementation improved sleep quality and sleep time [46,47,48]. Similarly, Chen and co-workers observed that oral melatonin administration in BC survivors improved sleep efficiency [49]. Deficiencies in the production or synthesis of endogenous melatonin have been found to be associated with the onset of many disorders like BC [50,51]. Sleep disorders are frequent in the general population, and cancer has been reported to activate sleep disorders itself [52,53]. In a recent meta-analysis, melatonin supplementation in BC patients had a positive effect on sleep quality [54]. However, it is rather controversial whether melatonin can be effective in combating anxiety and depression in cancer patients. Several studies have found that melatonin does not affect mood or cognitive function [46,47,49,55,56]. On the other hand, a meta-analysis by Fan and co-workers indicates that melatonin has a moderate effect on depression [57]. Similarly, other studies and controlled trials have found that melatonin soothes depressive symptoms [58,59]. Patients taking agents for sleeping disorders were not recruited in the present trial; therefore, the fatigue improvement in patients from the intervention group was probably attributable to melatonin supplementation.

Taking all these outcomes together, the effect of melatonin supplementation on CRF could be beneficial; however, there is a need to design and recruit larger cohorts in different oncology centers in order to investigate the association between melatonin and CRF, with or without lifestyle interventions such as MD implementation and body weight loss.

We are aware that our study has some limitations. The small number of trial participants and the single-hospital setting were important restrictions that could have affected the trial’s outcomes. In addition, all data regarding CRF were self-reported. Nevertheless, all appointed researchers in this study were well experienced and able to identify discrepancies and ask participants for further clarification. In order to avoid study bias, strict inclusion and exclusion criteria were implemented, while simple randomization was followed throughout the study. Patient allocation to the intervention and placebo groups was blind to all appointed researchers and was not revealed before data analysis.

## 5. Conclusions

In the present 3-month, randomized, placebo-controlled trial, patients with BC who underwent chemotherapy received 1 mg of melatonin oral supplement on a daily basis, along with a personalized dietary plan based on the MD. At baseline, CRF was positively associated with BMI and negatively associated with MedDietScore. At the end of the 3 months, patients in the melatonin group had significantly improved CRF compared to baseline, although no differences were observed with the placebo group, who only followed the MD. Larger intervention studies recruiting different oncology centers for longer periods of time could shed light on the potential associations between melatonin intake, lifestyle change, and CRF and on the underlying mechanisms of action as well.

## Figures and Tables

**Table 1:** Chemical composition of melatonin and placebo tablets.

	Active Ingredient	Excipients
Melatonin tablet	melatonin, 1 mg	mannitol, corn starch, stearic acid, ethyl cellulose, microcrystalline cellulose, magnesium stearate, sodium carboxymethylcellulose, wild berry flavor, silicon dioxide, sucralose.
Placebo tablet	-	dextrose, magnesium stearate, microcrystalline cellulose, berry flavor.

**Table 2:** Baseline characteristics of enrolled BC patients.

Characteristics	Enrolled Patients (*N* = 49)	Melatonin Group (*N* = 23)	Placebo Group (*N* = 26)	*p*-Value
Age (years); median (IQR)	52.0 (17.5)	54.0 (21.0)	51.0 (17.3)	0.873
Body weight (Kg); median (IQR)	69.9 (24.4)	69.9 (22.4)	70.7 (25.0)	0.582
BMI (kg/m^2^); median (IQR)	26.5 (9.4)	26.4 (8.1)	28.3 (11.4)	0.515
<18.5; N (%)	2 (4.1)	2 (8.7)	0 (0.0)	-
18.5–24.9; N (%)	18 (36.7)	8 (34.8)	10 (38.5)	-
25–29.9; N (%)	12 (24.5)	7 (30.4)	5 (19.2)	-
>30; N (%)	17 (34.7)	6 (26.1)	11 (42.3)	-
Fat mass %; median (IQR)	43.5 (15.3)	39.9 (17.7)	44.0 (14.2)	0.155
WC (cm); median (IQR)	96.0 (18.0)	96.5 (15.0)	96.0 (24.3)	0.588
Blood markers				
WBC (103/uL); median (IQR)	6.0 (3.0)	5.6 (2.4)	6.5 (4.8)	0.346
NEU %; median (IQR)	64.8 (16.0)	65.1 (17.9)	64.4 (14.7)	0.719
PL (103/uL); median (IQR)	227.0 (81.0)	199.0 (77.0)	243.5 (80.5)	0.602
HGB (g/dL); median (IQR)	11.8 (1.8)	11.8 (2.1)	11.8 (1.8)	0.846
HCT %; median (IQR)	35.7 (3.1)	35.7 (3.8)	35.8 (3.4)	0.938
LDH (u/L); median (IQR)	218.0 (115.0)	201.0 (136.4)	226.5 (128.7)	0.502
GLU (mg/dL); median (IQR)	98.0 (11.0)	97.0 (16.0)	98.0 (9.8)	0.484
ATC category				
A03F; N (%)	1 (2.0)	1 (4.3)	0 (0.0)	-
A02B; N (%)	10 (20.4)	4 (17.4)	6 (23.1)	-
H03A; N (%)	15 (30.6)	6 (26.1)	9 (34.6)	-
R03A; N (%)	2 (4.1)	1 (4.3)	1 (3.8)	-
H02A; N (%)	33 (67.3)	16 (69.6)	17 (65.4)	-
A04A; N (%)	29 (59.2)	15 (65.2)	14 (53.8)	-
L02B; N (%)	7 (14.3)	3 (13.0)	4 (15.4)	-
C08D; N (%)	1 (2.0)	1 (4.3)	0 (0.0)	-
B01A; N (%)	4 (8.2)	2 (8.7)	2 (7.7)	-
C07A; N (%)	3 (6.1)	1 (4.3)	2 (7.7)	-
C10A; N (%)	5 (10.2)	2 (8.7)	3 (11.5)	-
A03A; N (%)	1 (2.0)	1 (4.3)	0 (0.0)	-
C08C; N (%)	1 (2.0)	1 (4.3)	0 (0.0)	-
C09C; N (%)	2 (4.1)	1 (4.3)	1 (3.8)	-
C10B; N (%)	1 (2.0)	0 (0.0)	1 (3.8)	-
C07C; N (%)	1 (2.0)	0 (0.0)	1 (3.8)	-
A10B; N (%)	1 (2.0)	0 (0.0)	1 (3.8)	-
C09D; N (%)	1 (2.0)	0 (0.0)	1 (3.8)	-
P01B; N (%)	1 (2.0)	0 (0.0)	1 (3.8)	-
Current smokers; N (%)	0 (0.0)	0 (0.0)	0 (0.0)	-
ECOG performance status				
Score 0; N (%)	28 (57.1)	15 (65.2)	13 (50.0)	-
Score 1; N (%)	21 (42.9)	8 (34.8)	13 (50.0)	-
MedDietScore; median (IQR)	33.0 (5.0)	32.0 (5.0)	34.5 (6.3)	0.379
FACIT-F scale; median (IQR)	123.0 (26.5)	122.0 (45.0)	127.0 (27.5)	0.161

Continuous variables are presented as median and interquartile range (IQR). Qualitative variables are presented with absolute (N) and relative frequencies (%). For the comparison between the two treatment groups, the Mann–Whitney rank test was used. Level of statistical significance was set at 0.05. BC, breast cancer; BMI, body mass index; WC, waist circumference; ECOG, Eastern Cooperative Oncology Performance Status; WBC, white blood count; NEU, neutrophils; PL, platelets; HGB, hemoglobin; HCT, hematocrit; LDH, lactate dehydrogenase; GLU, glucose; ATC, Anatomical Therapeutic Chemical; FACIT-F, Functional Assessment of Chronic Illness Therapy—Fatigue.

**Table 3:** Regression analysis addressing associations of FACIT-F total score with BMI and MedDietScore.

Tested Association	Unadjusted Model		Adjusted Model 1		Adjusted Model 2		Adjusted Model 3	
	*Beta*	*p*-Value	*Beta*	*p*-Value	*Beta*	*p*-Value	*Beta*	*p*-Value
FACIT-F								
BMI	−0.304	**0.034**	−0.300	**0.028**	−0.509	**0.041**	−0.882	**0.003**
MedDietScore	0.389	**0.006**	0.337	**0.019**	0.338	**0.019**	0.248	0.179

All values are log-transformed. Level of statistical significance was set at *p* ≤ 0.05. Bold p-values represent statistically significant associations between variables. Model 1: adjusted for age. Model 2: adjusted for age and WC. Model 3: adjusted for age, WC, WBC, PL, LDH, GLU. BMI, body mass index; WC, waist circumference; WBC, white blood count; PL, platelets; LDH, lactate dehydrogenase; GLU, glucose; FACIT-F, Functional Assessment of Chronic Illness Therapy—Fatigue.

## Data Availability

Data are unavailable due to privacy restrictions.

## Supplementary Materials

The following supporting information can be downloaded at: https://www.mdpi.com/article/10.3390/cancers16040802/s1, Table S1: Inter- and intragroup comparisons regarding anthropometry, blood markers, MedDiet and FACIT-F scores.
