# Supplementary material for: The Effect of Melatonin Supplementation on Cancer-Related Fatigue during Chemotherapy Treatment of Breast Cancer Patients: A Double-Blind, Randomized Controlled Study
Source: Cancers (Basel). 2024 Feb 16;16(4):802. doi: 10.3390/cancers16040802 (PMC10887218; doi:10.3390/cancers16040802)
Supplement: Supplementary file 1 [file cancers-16-00802-s001.zip › cancers-2857460-supplementary.pdf]

Supplementary Table S1. Inter- and intragroup comparisons regarding anthropometry, blood markers, MedDiet and FACIT-F scores

Characteristics		Melatonin Group (*N* = 23)		*p-value**	Placebo Group (*N* = 26)		*p-value**	*p-value†*
		before	after		before	after		
Anthro-pometry:	BW (kg)	69.9 (22.4)	68.0 (19.1)	**0.008**	70.7 (25.0)	68.5 (23.4)	**< 0.001**	0.602
	BMI (kg/m²)	26.4 (8.1)	25.6 (6.6)	**0.012**	28.3 (11.4)	28.0 (11.2)	**< 0.001**	0.483
	Fat mass %	39.9 (17.7)	39.0 (12.0)	**0.042**	44.0 (14.2)	43.0 (13.2)	**< 0.001**	0.061
	WC (cm)	96.5 (15.0)	94.0 (16.5)	**0.005**	96.0 (24.3)	95.0 (23.3)	**< 0.001**	0.423
Blood markers:	WBC (103/uL)	5.6 (2.4)	5.0 (4.2)	0.345	6.5 (4.8)	6.3 (3.8)	0.136	0.346
	Neutrocytes %	65.1 (17.9)	63.0 (14.0)	0.859	64.4 (14.7)	65.0 (9.6)	0.573	0.719
	PL (103/uL)	199.0 (77.0)	231.0 (93.0)	0.443	243.5 (80.5)	242.0 (115.0)	0.616	0.602
	HGB (g/dL)	11.8 (2.1)	11.9 (1.0)	0.802	11.8 (1.8)	11.9 (2.0)	0.782	0.846
	HCT %	35.7 (3.8)	35.2 (4.9)	0.836	35.8 (3.4)	36.2 (3.7)	0.898	0.938
	LDH (U/L)	201.0 (136.4)	227.0 (108.0)	0.426	226.5 (128.7)	220.0 (67.0)	0.339	0.502
	Glu (mg/dL)	97.0 (16.0)	97.0 (8.0)	0.777	98.0 (9.8)	97.0 (13.0)	0.306	0.484
MedDiet:	MedDiet Score	32.0 (5.0)	34.0 (6.0)	**0.004**	34.5 (6.3)	35.0 (5.0)	**< 0.001**	0.165
FACIT-F scores:	Total Score	122.0 (45.0)	128.0 (32.0)	**< 0.001**	127.0 (27.5)	132.5 (28.5)	0.610	0.452
	PWB	20.0 (7.0)	21.0 (3.0)	**0.003**	20.5 (6.3)	22.0 (6.3)	0.976	0.658
	SFWB	20.0 (5.0)	22.0 (7.0)	**0.007**	23.0 (8.0)	23.5 (5.8)	0.366	0.444
	EWB	20.0 (7.0)	22.0 (6.0)	**0.002**	21.0 (8.3)	22.0 (7.0)	0.475	0.630
	FWB	20.0 (8.0)	22.0 (7.0)	**0.001**	20.0 (3.0)	20.0 (4.5)	0.253	0.513
	Fatigue	40.0 (18.0)	41.0 (16.0)	**0.026**	43.0 (5.5)	44.0 (7.3)	0.104	0.136

Data are expressed as median and interquartile range (IQR). *p-value: significant differences between baseline and follow up (3 months) analyzed by the Wilcoxon signed-rank test; †p-value: significant differences between the Placebo and the Melatonin group at follow up analyzed by Mann–Whitney rank test. Level of statistical significance was set at 0.05. BC, breast cancer; BMI, body mass index; WC, waist circumference; WBC, white blood count; NEU, neutrophils; PL, platelets; HGB, hemoglobin; HCT, hematocrit; LDH, lactate dehydrogenase; GLU, glucose; FACIT-F, Functional Assessment of Chronic Illness Therapy – Fatigue; PBW, physical well-being; SFWB, social/family well-being; EWB, emotional well-being; FWB, functional well-being
